# Bacillus Tuberculosis

**Published:** 1883-06

**Authors:** 


### Bacillus Tuberculosis.

For a very small subject, the bacillus has come to occupy a very large space in medical literature. We already have published several original papers which have generally supported the theories so ably advanced by Koch. The present number contains an excellent article, entitled "Contagium Animatum," to which we would direct the attention of our readers. There has been, however, no lack of able controversionalists, who have undertaken the refutation of the etiological relation of the bacillus with tuberculosis. In England (a country always conservative in medical as in other affairs), the majority of the profession have denied the parasitic origin of phthisis, and one of the physicians in the "Brompton hospital for consumptives," affirms, that after seventeen years' experience, he had never seen a case of infection, directly or indirectly. The "bacillians," however, have scored a point, by the report to the "Association for the advancement of medicine by research," on the relation of micro-organisms to tuberculosis. It is made by Dr. Watson Cheyne, whose name in England carries much weight, as he is well known as an earnest, thorough and conscientious observer and student. The April number of the *London Practitioner*, devoted its entire eighty pages to the report, which is well worthy of a careful perusal, though too long for republication in our pages. We hope, in a subsequent number, to reproduce portions of it. The "vital points" in it may be summed up as follows:

That tuberculous processes in the lungs are due to the tubercle bacilli, and to them only.

That the bacilli are present in the intestinal ulceration, which often occur in phthisis, and which are supposed to be due to swallowing sputum.

That milk of tuberculous cows may be infectious.

That the absolute diagnostic mark of a tuberculous process is the presence of the bacilli.

The heredity of tubercles is denied.

He gives the following as the sequence of events in phthisis: The bacilli which reach the lungs by inhalation, develop in the epithelial cells lining an alveolus; this alveolus becomes filled with cells; neighboring alveoli become infected, and the same process goes on in them. The further result will depend on the number and growth of the bacilli, and whether the patient is a good soil for their development. If they develop well, we have caseous pneumonia; if they grow slowly and with difficulty, we have fibroid phthisis. In the former case, the alveoli become distended early with epitheliod cells; this leads to inflammation of the walls of the alveoli; the cells soon undergo cheesy degeneration, and the presence of the masses lead to atrophy or sloughing of the alveoli. Infection of the neighboring parts of the lung occurs, both by continuity, and also by partial coughing up and re-inhalation of the bacilli into other parts of the lung. In the case of fibroid phthisis, the bacilli are few and grow only with difficulty. Thus fibrous formation occurs extensively. In parts, the process may be more rapid, and there cheesy masses are formed, which may lead to breaking down of the lung and the formation of cavities. Dr. Cheyne affirms also that the bacilli may be found in the sputum before any diagnosis can be made out by physical signs, and refers to the methods devised by Koch, Ehrlich, Baumgarten, and others, for demonstrating their presence, most of which have already appeared in this journal.

